# Novel Hybrid Manufacturing Process of CM247LC and Multi-Material Blisks

**DOI:** 10.3390/mi11050492

**Published:** 2020-05-12

**Authors:** Xiqian Wang, Luke N. Carter, Nicholas J. E. Adkins, Khamis Essa, Moataz M. Attallah

**Affiliations:** 1 School of Metallurgy and Materials, University of Birmingham, Birmingham B15 2TT, UK; wangxiqian513@gmail.com (X.W.); l.n.carter@bham.ac.uk (L.N.C.); adkins_nick@hotmail.com (N.J.E.A.); 2 School of Engineering, University of Birmingham, Birmingham B15 2TT, UK

**Keywords:** selective laser melting, hot isostatis pressing, hybrid manufacturing, CM247LC, nickel superalloys, multi-material, blisks

## Abstract

The study on CM247LC used the traditional approach for Near-Netshape Hot Isostatic Pressing (NNSHIP) with sacrificial low carbon steel tooling, which was built using Selective Laser Melting (SLM), to produce a shaped CM247LC blisk. The assessment of the microstructure focused on both the exterior components in order to determine the depth of the Fe-diffusion layer and on the interior microstructure. Samples were extracted from the Hot Isostatic Pressed (HIPped) components for tensile testing at both room and elevated temperatures. The components were scanned to assess the geometrical shrinkages due to Hot Isostatic Pressing (HIPping). An oversized blisk was also produced based on the measurements as a demonstrator component. In addition, a further study was carried out on a novel idea that used a solid IN718 disk in the centre of the blisk to create a multi-material component.

## 1. Introduction

Additive Manufacturing (AM), as a netshape technology, has developed rapidly in the past 10 years. Recent interest in the processing of more expensive materials has been driven by the ability of the technique to potentially reduce material waste [1,2,3,4]. Selective Laser Melting (SLM) is an AM process that uses a high intensity laser to selectively melt defined areas geometries layer by layer and therefore allows the production of near netshape components directly from the metal powder [5,6,7,8,9]. 

CM247LC is a chemically modified version of the more common MAR M247. Table 1 shows the chemical composition of CM247LC, which was designed with a low C-content [3]. Furthermore, the improved performance of CM247LC is due to the modified elements from MAR M247 [10,11,12]. Therefore, the castability, alloy ductility, fatigue strength and carbide stability of CM247LC DS have been improved compared with MAR M247 [13]. Furthermore, CM247LC DS samples possess the highest tensile strength at a various range of temperatures compared with IN6203 DS and IN792 DS samples [14].

Work presented in a previous study investigated SLM of the high temperature Ni-superalloy CM247LC [15]. It was found that CM247LC showed high susceptibility to cracking due to the high γ’ volume fraction during processing and that a HIPping post-processing step was required to reduce and heal the defects, especially the cracks and any residual porosity, within the SLM-processed samples. Post-SLM heat treatments produced a coarse grain structure, yet still with very anisotropic mechanical properties with respect to the build orientation.

SLM is capable of producing components with complex shapes, but they are limited by relatively low building rates. In contrast, HIPping shows an advantage in the processing rate, although it requires capsules (female moulds) to be machined for powder HIPping [16,17,18]. Therefore, a hybrid route for manufacturing Ti-6Al-4V, combining SLM and HIPping, was suggested by Das et al. [19] under the name ‘in-situ shelling’, and was recently further developed by incorporating modelling [20,21]. This approach involves manufacturing autogenous tooling using SLM, which gets concurrently filled with powder during SLM and then Hot Isostatic Pressed (HIPped) [20,21]. However, there is no literature on SLM/HIP of Ni-superalloys. Additionally, Das et al. raised the issue of oxygen pickup during processing of Ti-alloys using the SLM/HIP route [19]. Other hybrid manufacturing routes that combines either SLM or HIPping with complimentary postprocessing techniques were also proposed but not in details [22,23,24,25,26]. The key benefits and novelty associated with this work include: the feasibility of creating multi-material complex structures through the incorporation of powder HIP diffusion bonding into a solid material, while using an additively manufactured canister to increase the geometrical complexity of the resulting structure; when a dissimilar material pair is used, it is possible to tailor the component design to the application, whereby (in the case of the blisk) the disk is made out of a fatigue resistant material (IN718), while the blades which are exposed to higher temperatures are made out of a high temperature alloy (CM247LC); since CM247LC cannot be processed using additive manufacturing due to its crack susceptibility, the use of SLM to manufacture tooling enables the fabrication of crack-susceptible metallic alloys, such as TiAl, high γ′ Ni-superalloys and Nb-based intermetallics, through the proposed indirect route.

## 2. Experimental Methods

Near-Netshape HIPping (NNSHIP) of a CM247LC blisk component using steel tooling built via SLM has been developed (denoted as Fe-tooling SLM/HIP). Table 2 summarises key stages alongside the components being designed, as well as the materials being fabricated.

### 2.1. Fabrication of Blisk

**Figure 1:**
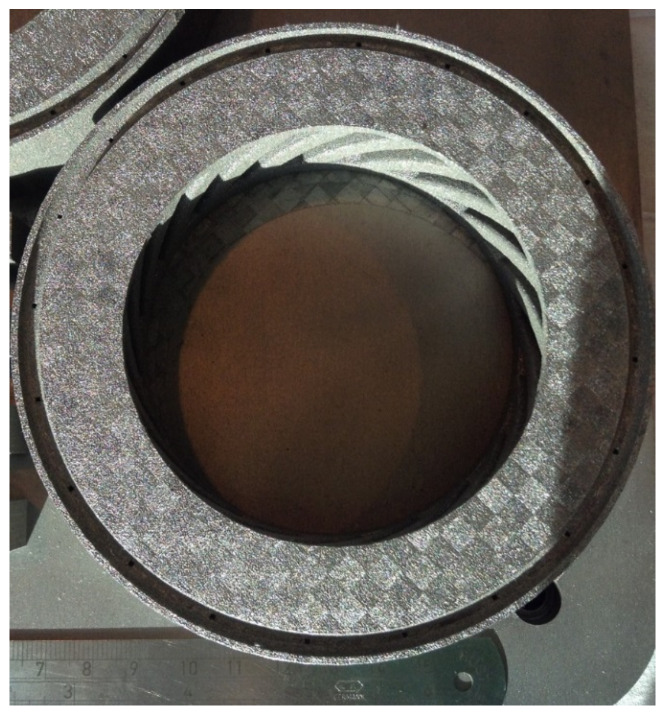
An image of Selective Laser Melting (SLM)-processed Fe blade tooling attached to the substrate.

**Figure 2:**
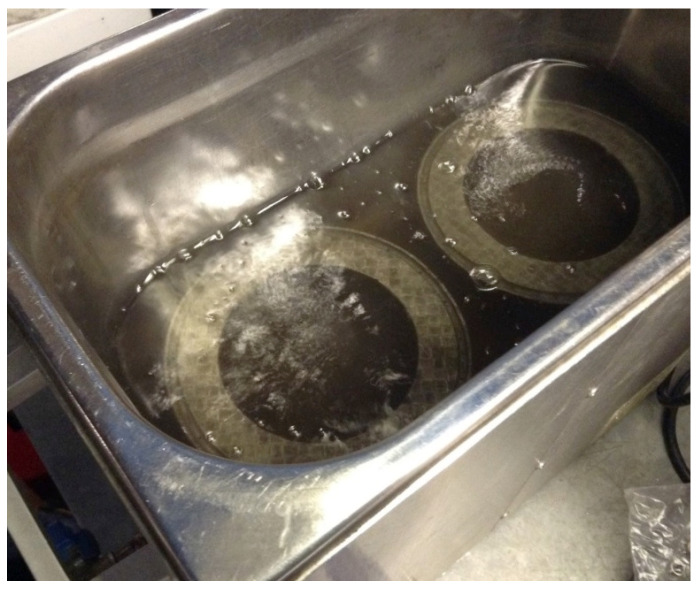
An image of degreasing SLM-processed blade tooling with acetone in an ultrasonic bath.

**Figure 3:**
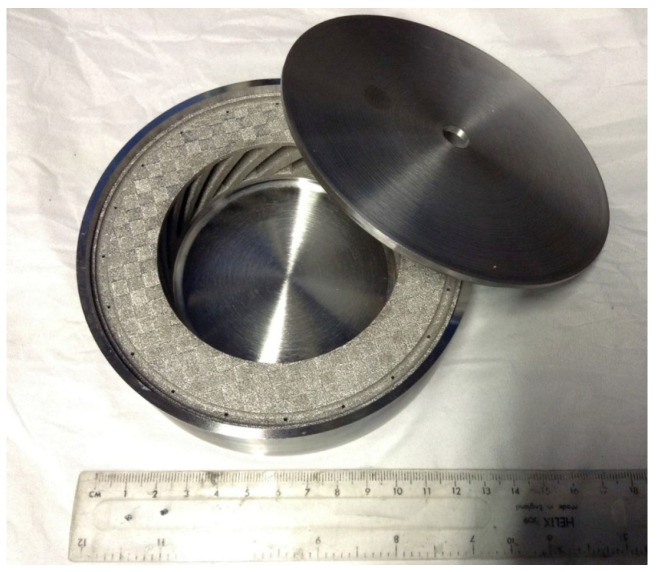
An image of three parts before assembling.

To fabricate Fe-tooling SLM/HIP of CM247LC blisks, there are six detailed steps:

a. The fabrication of blade tooling using an SLM process with Fe powder, Figure 1. To speed up building rates, processing conditions with a fast scan speed were selected (3000 mm/s at 300 W laser power). These conditions also had the benefit that the production of large amounts of black soot during processing was much reduced.
b. The components were sprayed with a thin layer of oil (WD40) before cutting from the substrate using Electrical Discharge Machining (EDM) to prevent rusting.
c. To avoid contaminating the powder, all of the parts, lid, base and SLM-processed tooling, were degreased with acetone in an ultrasonic bath before assembling, Figure 2.
d. The assembled parts were welded together with a filling tube, Figure 3.
e. After vacuum testing, the assembled parts were filled with CM247LC powder with a size range of 0–150 µm. The container was filled on a vibrating table to ensure good filling of the cavities and high, reproducible packing density. The filled can was degassed overnight and then sealed by crimping and welding. The sealed can was then HIPped at 1260 °C for 2 h.
f. The HIPped component was scanned with a Faro laser scanner.
g. The outer Fe-tooling was removed by pickling in nitric acid starting with concentrated acid (50%) for a day, diluted acid for 2–3 days and more dilute acid for 2 more days.

### 2.2. Fe-tooling SLM/HIP of CM247LC Blisk with IN718 Inserted Disk

**Figure 4:**
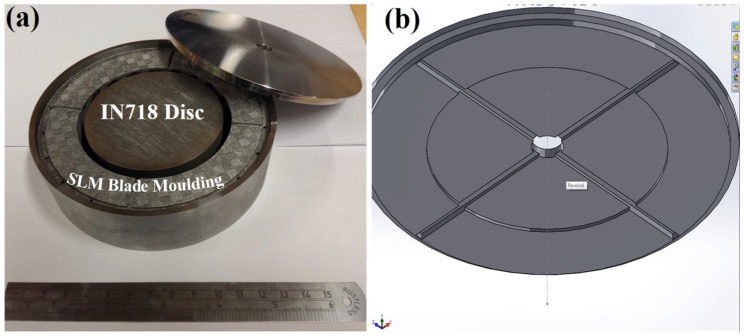
(**a**) An image of the parts including IN718 inserted disk; (**b**) Computer-aided Design (CAD) model of a modified can lid.

An additional study was carried out on a novel idea that uses a solid IN718 disk in the central blisk sections replacing much of the CM247LC powder, as shown in Figure 4a. The IN718 disk was cut from an ingot using EDM, and the recast layer was removed by pickling prior to assembly. The top lid was also further modified to improve degassing by adding extra grooves to locate the disc inside the can using a 1 mm deep step in the centre of the lid, seen in Figure 4b.

### 2.3. Surface Finish

The surface finish of the blades was assessed using a profilometer (Ambios, XP-200, Ashprington, UK). As each cavity of the blade is inclined at 45° without any support structures during SLM processing, the sides of the blades have different surface finishes after SLM processing and HIPping. The measurement of surface roughness was carried out on both sides with 3 mm measuring distance. The roughness reported was the average of 3 measurements.

### 2.4. Microstructural Observation

For Fe-tooling SLM/HIP of CM247LC, a study of Fe diffusion was carried out to assess the processing parameters and to determine the allowance that will be required for machining. Samples were cut both from the HIPped can prior to and after the pickling followed by grinding and polishing for microstructural observation. The microstructural observation was carried out using a Jeol7000 Scanning Electron Microscope (SEM), fitted with backscattered and Energy Dispersive X-ray Spectroscopy (EDS) detectors. One of the blades was also cut from the component to observe the microstructures and assess consolidation after HIPping, especially at the thinnest part of the blade.

### 2.5. Mechanical Testing

For Fe-tooling SLM/HIP of CM247LC, both Room Temperature (RT) and Elevated Temperature (ET) tensile tests at 760 °C ± 3 °C were performed (Westmoreland Mechanical Testing & Research, Limited) to assess the influence of the HIP processing parameters determined in this study. For the ET tests, the heating up time was approximately 60 mins and 30 mins for soaking time. Rod bars (measuring ∅ = 8 mm, length = 55 mm) were cut from the HIPped and pickled blisk followed by machining according to ASTM E8-15a and ASTM E21-09 [27]. The bond line (between the powder and the solid part) was centred in the gauge length of the sample. All specimens were tested in strain rate controlled at 0.005 strain/min beyond yield at which point 0.05 strain/min was adopted and the extensometer was removed. 

## 3. Results and Discussion

**Figure 5:**
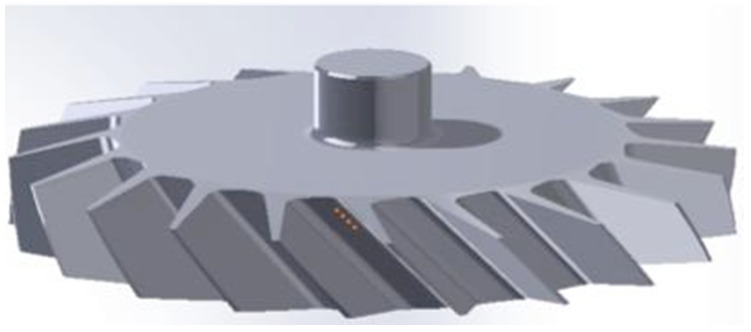
CAD model of the demonstrator geometry for a blisk.

**Figure 6:**
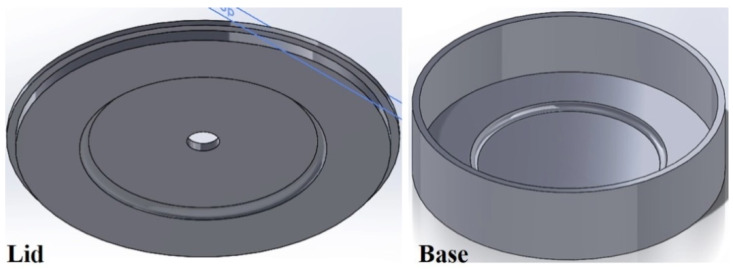
CAD model of the final version of the simple ‘over can’ design.

**Figure 7:**
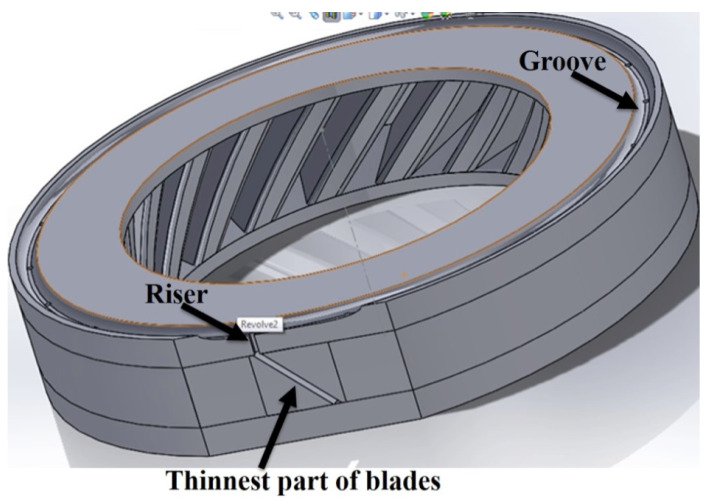
CAD model of the final version of the SLM-processed blade tooling.

As for SLM/HIP of CM247LC, the work was performed using SLM to create a mild steel tooling (negative mould) of a blisk, Figure 5, whereby the sacrificial mild steel tooling, involved in the production of a CM247LC SLM/HIP blisk, consists of a simple ‘over-can’ made by machining and the complex geometry SLM-processed blade tooling. The final version of the can and the blade tooling are shown in Figure 6 and Figure 7. It is important that the can is filled completely, including the narrow blade sections shown in Figure 5. The final blade tooling was designed to have an extension, similar to a riser in casting, to ensure that each blade cavity is completely filled with powders.

**Figure 8:**
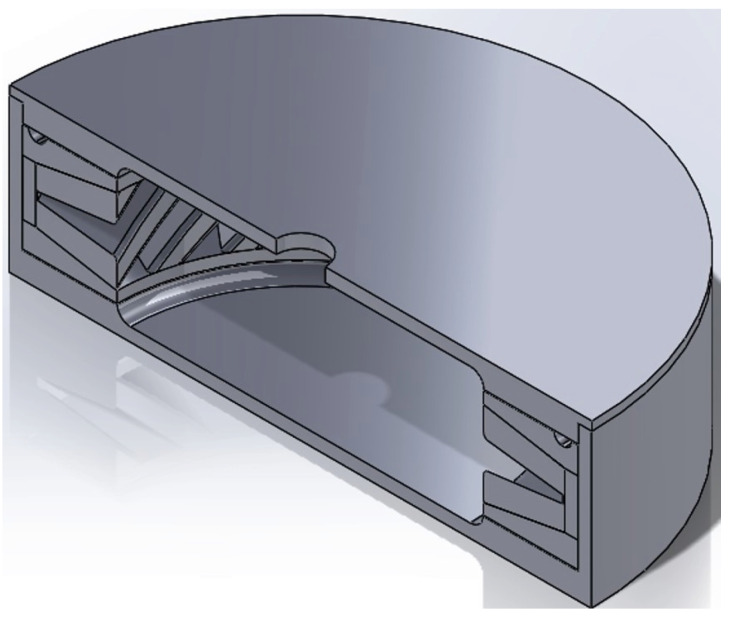
CAD model of the assembled final can design.

The ‘riser’ was located at the thinnest part of the blade, with one for each blade, to ensure each cavity was filled with powder evenly; an extra groove ring was added to connect the ‘risers’ to provide a small powder reservoir to feed the cavities if required, as seen in Figure 7. The final assembled can design is shown in Figure 8. The filling tube was added at the welding stage.

### 3.1. The 1st Blisk

**Figure 9:**
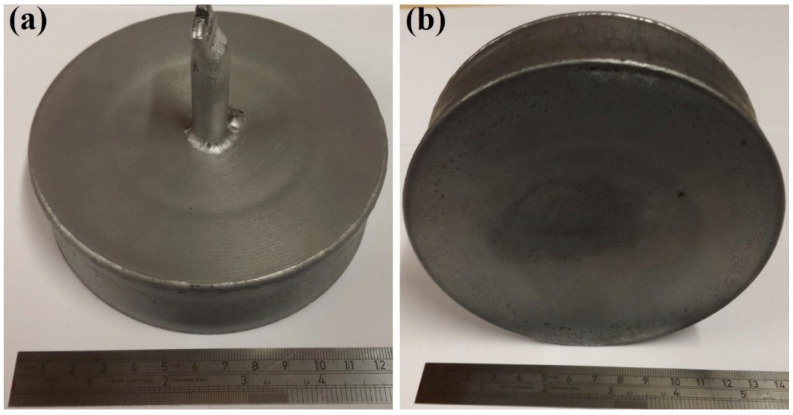
Images of the powder canister after HIPping: (**a**) top view of the component, showing a shallow sink mark in the middle part of the component where the disk is located; (**b**) the bottom side of the canister showing even shrinkages towards to the centre and also a shallow sink mark on the bottom of the canister.

**Figure 10:**
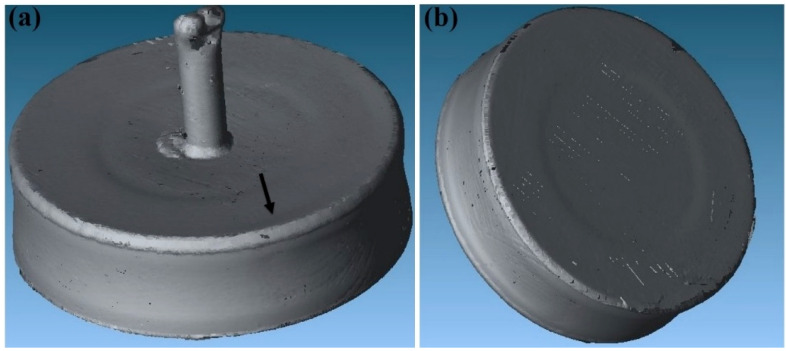
Images of the components after HIPping, as scanned using a Faro laser scanner: (**a**) top view of the component showing a shallow sink mark in the middle part and a small concave area indicated in the image due to less powder filled into the groove; (**b**) clearer contraction evenly towards the centre of the components and a shallow sink mark on the bottom of the canister.

The detailed steps for fabricating Fe-tooling SLM/HIP CM247LC blisks were presented in the Experimental Methods section. After HIPping, from both top and bottom, a very shallow dip was apparent in the middle part of the component, where the disk formed after HIPping; the side of the component also shrank slightly towards the centre evenly from each direction, as shown in Figure 9. The HIPped component was scanned with a Faro laser scanner, as seen in Figure 10. It showed a small concave area close to the edge due to poor filling inside the groove, but the rest of groove showed no concave area.

**Figure 11:**
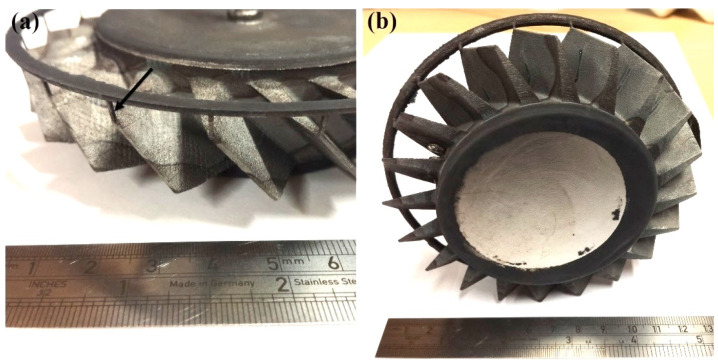
The image of the final Fe-tooling SLM/HIP blisk after pickling, showing (**a**) the fine powder ‘riser’ structure pointed to by an arrow and (**b**) consolidated into CM247LC pins after HIPping.

After pickling using nitric acid, the steel tooling was removed and the netshape blisk was produced using Fe-tooling SLM/HIP, as shown in Figure 11. The fine risers were all filled with powder and became solid CM247LC pins connecting the blades and groove ring. The top and bottom of the disk sections were near to flat. Therefore, the can, including each blade, was filled with powder successfully before HIPping and consolidated into the complete blisk without any defects.

### 3.2. The 2nd Oversized Blisk

**Figure 12:**
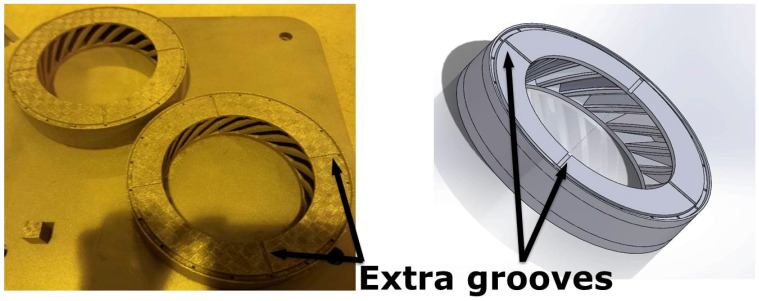
The image and CAD model of second oversized SLM-processed blade tooling.

One of the concerns associated with HIPping is the need to consider the degree of shrinkage associated with powder consolidation. To assess the shrinkage of the first Fe-tooling SLM/HIP blisk due to HIPping, sectioned profiles were exported from 3D scanning GOM inspection software and measured in Solidworks. To accommodate shrinkages and machining, the second oversized SLM-processed blade tooling was designed based on sizes listed in Table 3. The size of the second tooling design is also limited by: (i) avoiding the need for a support structure within the blades during SLM processing; (ii) the diameter of the simple mild steel ‘over-can’ (limited by the size of the HIP vessel). The degassing of the first blisk was slow and therefore extra grooves were added on the top of the second (oversized) blade tooling, as shown in Figure 12.

**Figure 13:**
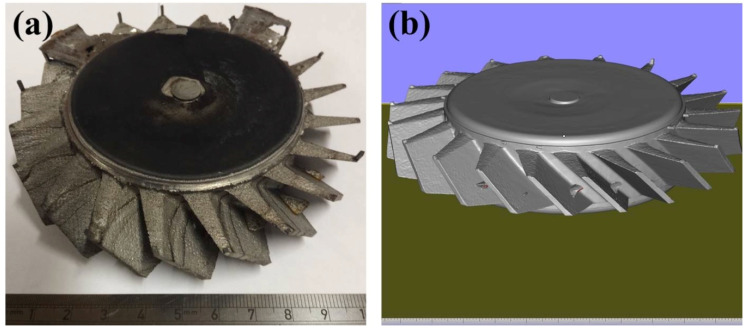
(**a**) The image of the second oversized Fe-tooling SLM/HIP blisk; (**b**) 3D image of the second oversized Fe-tooling SLM/HIP blisk scanned using a GOM optical 3D scanner.

The second (oversized) blisk was successfully fabricated, as shown in Figure 13a. Although it was entirely produced from powder like the second blisk, the top and bottom of the disk were nearly completely flat. A further GOM optical 3D scan was carried out, as shown in Figure 13b. It can be clearly seen that the blades are thicker than the first blisk, shown in Figure 11.

### 3.3. Blisk with IN718 Inserted Disk 

**Figure 14:**
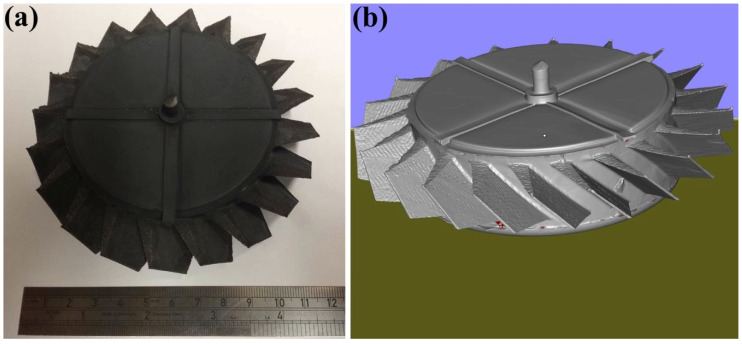
(**a**) Image of Fe-tooling SLM/HIP of CM247LC blisk with IN718 inserted solid disk; (**b**) 3D image of Fe-tooling SLM/HIP of CM247LC blisk with IN718 inserted solid disk scanned using GOM scanning.

To assess the utility of this approach for making multi-material/alloy structures, Fe-tooling SLM/HIP of a CM247LC blisk with an IN718 inserted solid disk was also attempted, as shown in Figure 14. The extra grooves filled with powder on the top of the component were revealed, which were added to the top lid to improve degassing and locate the IN718 disk inside the can.

### 3.4. Surface Finish

As each cavity of the blade is inclined by 45° without any support structures during SLM processing, as such the sides of the blades have different surface finishes. The measurement of surface roughness was carried out on both sides with 3 mm measuring distance. The roughness was averaged over 3 measurements. The rougher surface showed an average Ra of 14.5 μm, whereas it was 6.5 μm on the smoother surface. 

### 3.5. Microstructural Assessment—Results and Discussion

#### 3.5.1. Blisk

**Figure 15:**
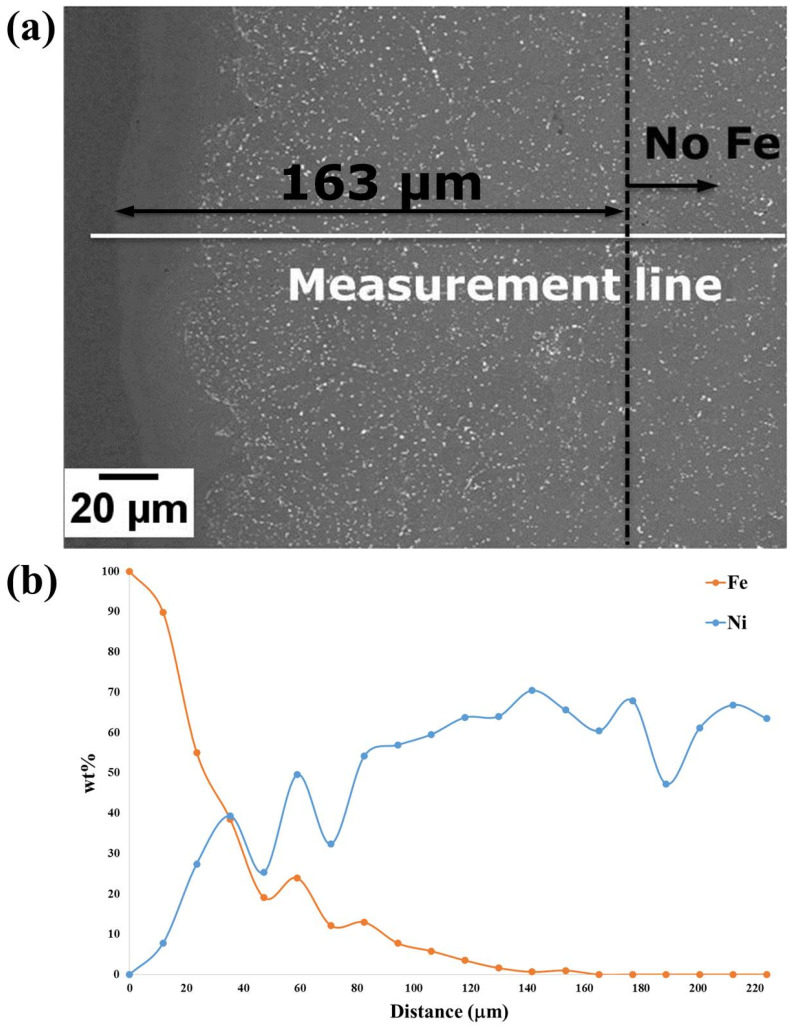
(**a**) BSE micrograph for the sample cut from the Fe-tooling SLM/HIPped can prior to pickling; (**b**) EDX line result along the measurement line indicated in (a); it is shown that Fe decreases to ~0 wt.% at around 163 μm.

**Figure 16:**
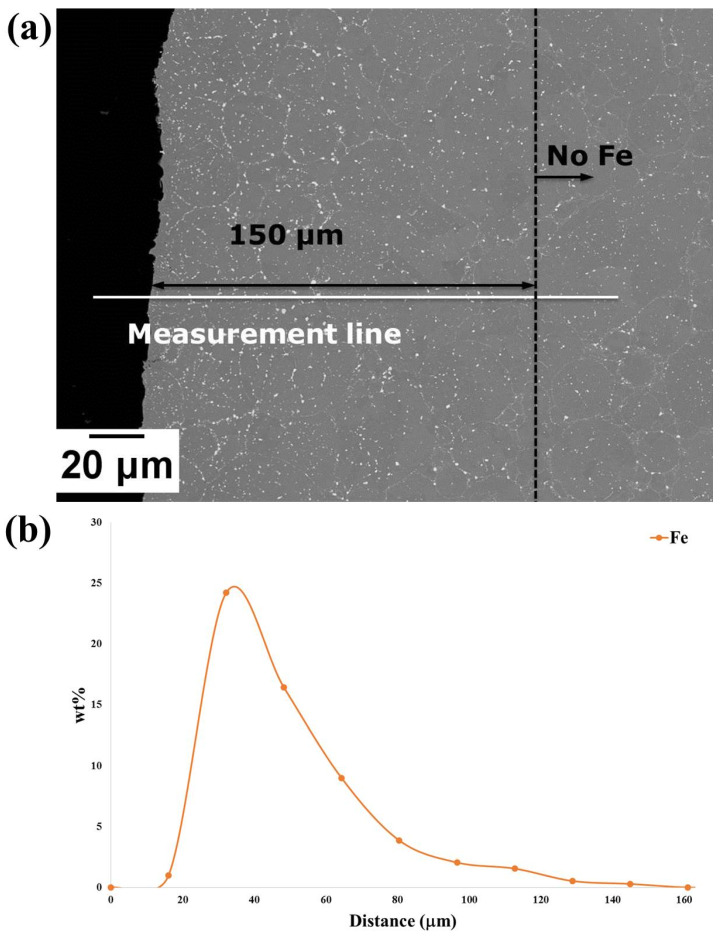
(**a**) BSE micrograph of the sample cut from the Fe-tooling SLM/HIPped blisk after pickling; (**b**) EDX line result along the measurement line indicated in (a); it is shown that Fe decreases to ~0 wt.% at around 150 μm.

**Figure 17:**
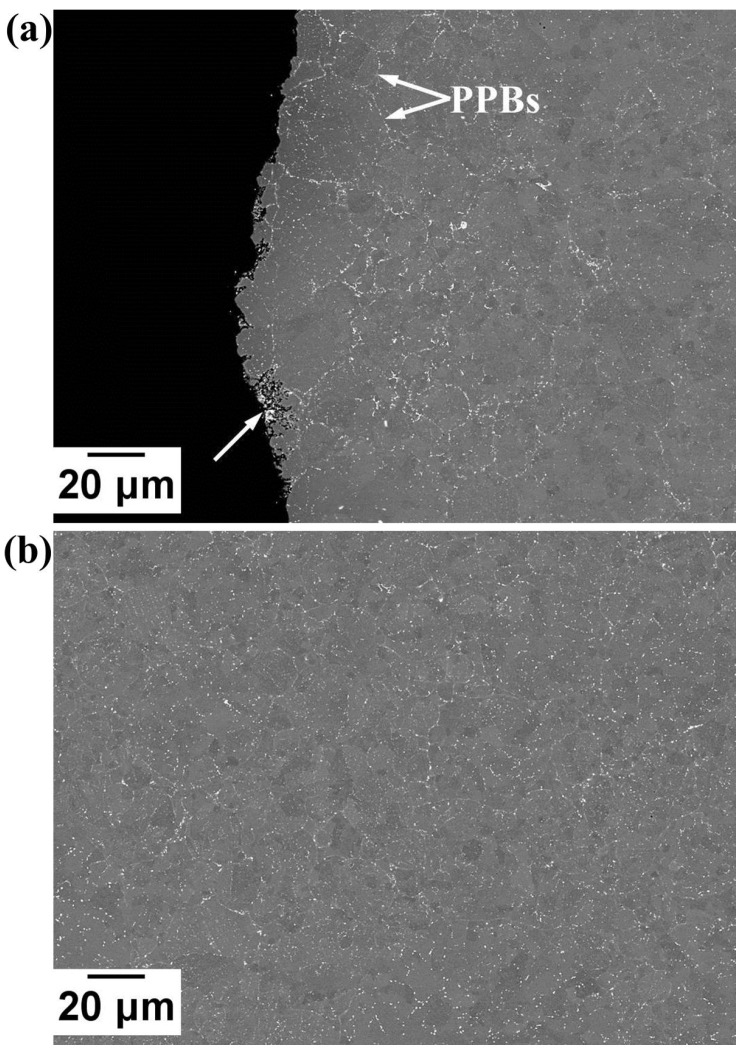
BSE micrographs showing (**a**) the thinnest part of the blade, where the nonconsolidated area is indicated by the white arrow; (**b**) further away from the edge of the blade.

A study of Fe diffusion from the sacrificial mild steel tooling was carried out to assess the depth of Fe-diffusion in order to determine the allowance that will be required for machining. It was found that the diffusion distance of Fe (measured using Energy-dispersive X-ray spectroscopy EDX) was ~163 μm before the removal of the steel can (by pickling), as shown in Figure 15. Following pickling, the Fe diffusion layer was ~150 μm, as shown in Figure 16. One of the blades was also cut from the component to observe the microstructure and assess its consolidation after HIPping focussing on the thinnest part of the blade. A limited area that was not fully consolidated was also observed; however, this was less than 10 μm away from the edge, as shown in Figure 17a, and would therefore be removed during finishing. Prior Powder-Particle Boundaries (PPBs) were also more visible close to the thin edge of the blade, seen in Figure 17a. This could be due to the slightly higher oxygen content left at the thinnest area during degassing, which may provide nucleation sites for oxy-carbides. Again, those observed near to the edge of material that would be removed in the final machining. Further away from the edge showed nearly 100% density with evenly distributed precipitates (Figure 17b). The diffusion of Fe requires machining of around 150 μm and could incorporate techniques to achieve the appropriate surface finish necessary for an aerodynamic component.

#### 3.5.2. Blisk with IN718 Inserted Disk

**Figure 18:**
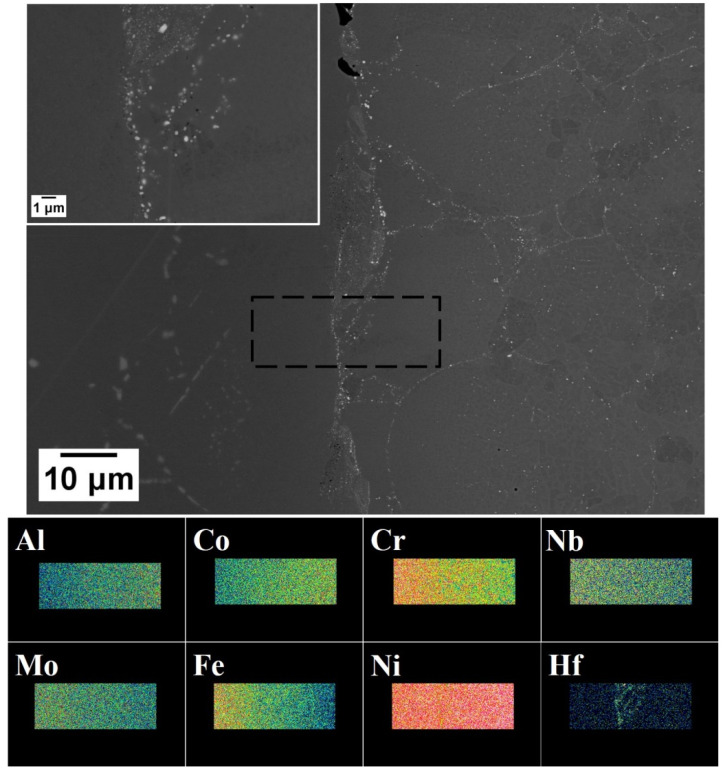
BSE micrographs of the boundary between the IN718 disk and the HIPped CM247LC powder, with a higher magnification BSE micrograph in the insert. X-ray maps of various elements are shown below the low magnification BSE.

A metallographic examination of the boundary between the IN718 disk and the HIPped CM247LC was carried out. There were high densities of Hf-rich precipitates (brighter particles on Back-scattered Electrons BSE) on the boundary (indicated by X-ray mapping) in Figure 18. It also showed that the chemical compositions varied cross the boundary, where Al and Co gradually increased from IN718 (left in BSE image) to CM247LC (right in BSE image), in contrast to Cr, Nb, Mo and Fe on X-ray maps, which decreased as seen in Figure 18. 

### 3.6. Mechanical Testing

**Figure 19:**
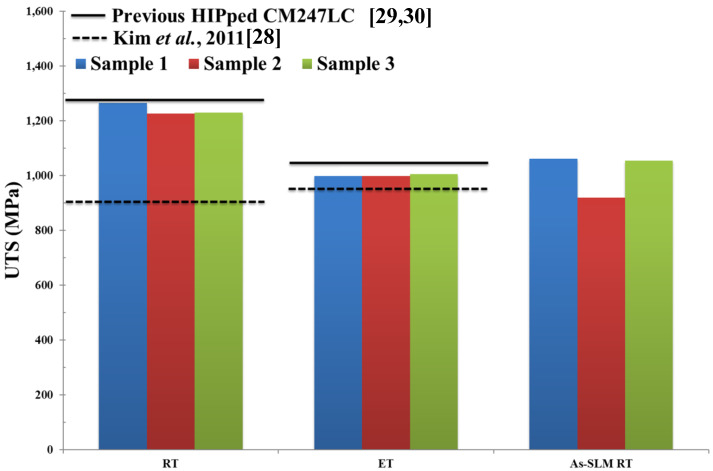
Bar plot showing Ultimate Tensile Strength (UTS) of HIPped CM247LC at Room Temperature (RT) and Elevated Temperature (ET), as well as SLM-processed CM247LC at RT for comparison [1]. Properties for conventionally cast and heat treated CM247LC are represented by the dashed line [28] and previous results of HIPped CM247LC undertaken in AMPLab by the solid line [29,30].

**Figure 20:**
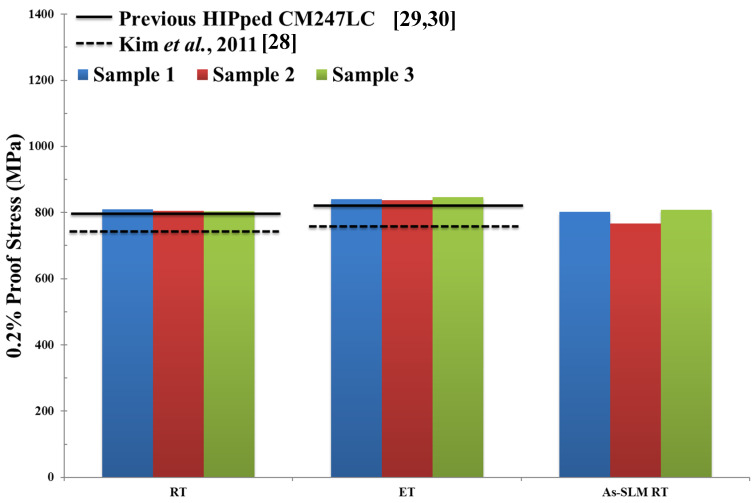
Bar plot showing 0.2% proof stress of HIPped CM247LC at RT and ET, as well as SLM-processed CM247LC at RT for comparison [1]. Properties for conventionally cast and heat treated CM247LC are represented by the dashed line [28] and previous results of HIPped CM247LC undertaken in AMPLab by the solid line [29,30].

**Figure 21:**
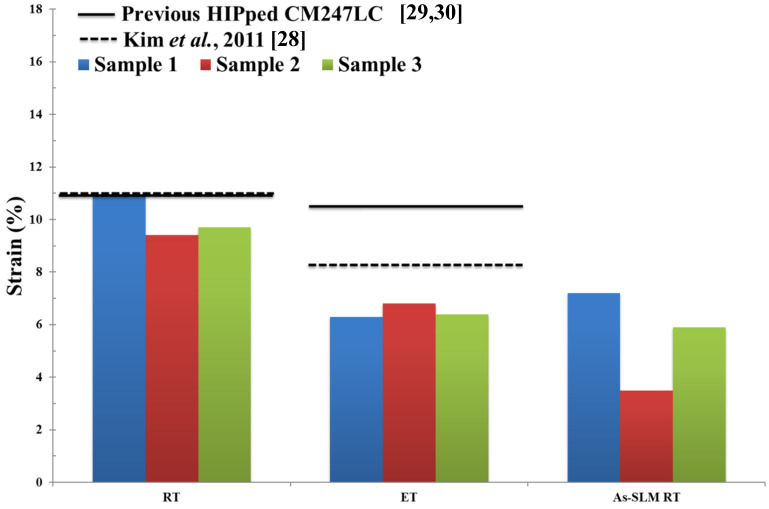
Bar plot showing % strain to failure of HIPped CM247LC at RT and ET, as well as SLM-processed CM247LC at RT for comparison [1]. Properties for conventionally cast and heat treated CM247LC are represented by the dashed line [28] and previous results of HIPped CM247LC undertaken in AMPLab by the solid line [29,30].

The Ultimate Tensile Strength UTS (MPa), 0.2% proof stress (MPa) and % strain to failure of Fe-tooling SLM/HIPped CM247LC at RT and ET are shown in Figure 19, Figure 20, Figure 21 respectively. Data from conventionally cast and heat treated CM247LC [28] and previous work on powder HIPping of CM247LC undertaken in AMPLab [29,30] as well as vertically SLM-processed CM247LC [1] are represented for comparison. The 0.2% proof stress of samples was marginally higher at ET than RT due to the ordered *γ*′ phase. On comparing the various processing routes investigated in this work, including the work undertaken previously [29,30] and in this paper, HIPped CM247LC showed higher UTS and 0.2% proof stress than conventionally cast and heat treated CM247LC at both RT and ET, and slightly poorer ductility due to the presence of PPBs [28]. Moreover, it had superior UTS and ductility to vertically SLM-processed CM247LC at RT. The previous powder HIPped CM247LC samples [29,30] were tested at 750°, which is slightly lower than that used in this study, therefore displaying higher ductility than Fe-tooling SLM/HIPped CM247LC.

## 4. Conclusions

Fe-tooling SLM/HIP of CM247LC was developed, and a can design which combines simple, mild steel ‘over-can’ tooling by machining with a complex blade tooling produced in low carbon steel by SLM successfully fabricated an oversized blisk. The initial component produced is of high quality. The HIPping parameters have been optimised based on an as-HIPped microstructure, the diffusion of Fe and mechanical properties. The Fe diffusion was less than 150 µm indicating the need for limited final machining. Compared with conventionally cast and heat treated CM247LC, HIPped samples show higher UTS and 0.2% proof stress at both RT and ET and slightly poorer ductility. The roughness of the blades’ surfaces was measured as 14.5 µm on the rougher side of the blades and 6.5 µm on the other side of the blades. A novel idea, Fe-tooling SLM/HIP of CM247LC blisk with IN718 inserted disk, was trialled and a hybrid component successfully produced. This is a potential area for future research as it has significant cost saving potential, with more effort required to improve the design of the interface between the two materials.

## Figures and Tables

**Table 1:** The chemical compositions of CM247LC (in wt.%) [3].

Cr	Co	Mo	W	Al	Ti	Ta	Hf	C	B	Zr	Ni
8.1	9.2	0.5	9.5	5.6	0.7	3.2	1.4	0.07	0.015	0.015	Bal

**Table 2:** Summary of key stages alongside components being designed in Selective Laser Melting/Hot Isostatic Pressing (SLM/HIPping) route.

Stages	Components	Materials	Comments
I	1st blisk	Fe and CM247LC powder	Assess shrinkages
II	2nd Oversized blisk	Fe and CM247LC powder	Attempt to achieve required sizes
III	Blisk with IN718 inserted disc	Fe, CM247LC powder and IN718 disc	Bring up a novel idea

**Table 3:** The size comparisons of different parts of blisk (dimensions in mm).

Parts		1st Tooling Design	Actual after HIP	Required	2nd Tooling Design
Blades Thickness	Thinnest	1.7	0.7-0.9	1.2	3.2
	Thickest	7.3	6.6	6.5	8.8
Blades Height	Lowest	17.4	16.4	17.6	19.5
	Highest	26.6	23.4	25.3	27.9
Blades Length	Shortest	17.6	17.1	14.8	17.8
	Longest	20	18.7	17.9	20.2
Disk	Dia.	82.4	74.9	82.4	84.4
